# COMPONENTS OF SUPPORTED DECISION-MAKING BY ADULT GUARDIANS FOR ADULTS UNDER GUARDIANSHIP IN JAPAN

**DOI:** 10.1093/geroni/igae098.3245

**Published:** 2024-12-31

**Authors:** Sachiko Kasahara

**Affiliations:** Shitennoji University, Habinko, Osaka, Japan

## Abstract

The adult guardianship system in Japan is designed to protect the rights and property of elderly individuals or those with diminished decision-making capacity. The system also allows for property management and personal care considerations. Adult guardians include relatives and non-relative guardians, but in Japan non-relative guardians account for over 80% of the total. For non-relative adult guardians who often meet the individual after being appointed as their guardian, respecting the individual’s wishes is an extremely important task in guardianship activities. The purpose of this research is to visualize the components of supported decision-making by non-relative guardians. The previous studies indicated that it was important to know what the specifics of supported decision-making for people under wards. The research was conducted from September to October of 2022 by self-administered questionnaires. The survey was mailed to 1,180 non-relative adult guardians in Japan. The data was collected Multistage sampling. The response rate was 25.2%. The statistical analysis was an exploratory factor analysis. Three factors in the specifics of supported decision-making were extracted by the factor analysis: Supporting for forming, expressing, and realizing of their wishes of the person under wards; Assessment and relationship with them; and Supporting by a team. The Cronbach’s alpha for internal consistency was greater than 0.8 for each factor. In addition, the three factors were associated each other. In conclusions, the present study found that the specifics of supported decision-making were Supporting their wishes as process, Preconditions for supported decision-making, and Supporting as a team.

